# Changes in hypsarrhythmia in West syndrome following combined high-dose prednisolone and vigabatrin therapy: A standardized, low-resolution, brain electromagnetic tomography study

**DOI:** 10.1097/MD.0000000000048017

**Published:** 2026-03-06

**Authors:** Jooyoung Lee, Ja Un Moon, Eu Gene Park, Il Han Yoo, Ji Yoon Han, Tae-Hoon Eom, Joong Hyun Bin

**Affiliations:** a Department of Pediatrics, College of Medicine, The Catholic University of Korea, Seoul, Republic of Korea.

**Keywords:** distributed source model, electroencephalography (EEG), source localization, standardized low-resolution brain electromagnetic tomography (sLORETA), West syndrome (WS)

## Abstract

This study aimed to quantitatively assess electrophysiological changes in patients with West syndrome (WS) treated with high-dose prednisolone and vigabatrin, using standardized low-resolution brain electromagnetic tomography (sLORETA). The study included 17 infants newly diagnosed with WS. Electroencephalography (EEG) recordings were obtained before treatment and again at 4 weeks after initiating combination therapy with oral prednisolone (8 mg/kg/day) and vigabatrin (100 mg/kg/day, increased to 150 mg/kg/day if needed). Artifact-free sleep EEG epochs were analyzed using sLORETA to estimate cortical current density across delta, theta, alpha, and beta frequency bands, with statistical comparisons performed using nonparametric voxel-wise permutation testing. At 4 weeks, 11 patients (64.7%) achieved complete cessation of spasms, and hypsarrhythmia resolved in 12 patients (70.6%). sLORETA analysis revealed significant reductions in cortical current density across all frequency bands, most prominently in the delta range. These reductions were most notable in the frontal cortex, particularly in the inferior and middle frontal gyri, with additional involvement of temporal and deep cortical regions, indicating an anterior-dominant, frequency-dependent treatment effect. The suppression of low-frequency activity aligns with attenuation of the pathological slow-wave components characteristic of hypsarrhythmia, indicating that combined high-dose prednisolone and vigabatrin therapy leads to a substantial reduction in cortical hyperexcitability. These findings provide quantitative neurophysiological evidence that treatment modulates dysfunctional cortical networks in WS. Furthermore, sLORETA can be a promising method for objectively monitoring treatment response and increasing the understanding of neurophysiological mechanisms underlying developmental and epileptic encephalopathies.

## 1. Introduction

West syndrome (WS) is the most common developmental and epileptic encephalopathy of infancy.^[1]^ The syndrome is defined by the classical triad of epileptic spasms, the distinctive electroencephalographic pattern of hypsarrhythmia, and developmental arrest or regression.^[2]^ WS affects approximately 0.16 to 0.42 per 1000 live births, with peak onset between 3 and 7 months of age, although cases may occur up to 24 months.^[3]^

Clinically, WS manifests as brief, bilaterally symmetric tonic spasms involving flexion or extension of the trunk, neck, and extremities. These spasms typically last only a few seconds and occur in clusters ranging from 20 to 100 episodes. The hallmark electroencephalography (EEG) feature, hypsarrhythmia, is characterized by chaotic, high-amplitude slow waves interspersed with multifocal spikes and sharp waves of varying amplitude, morphology, and distribution. Affected infants may exhibit developmental plateau or regression, with hypotonia, abnormal primitive reflexes, lethargy, and poor reactivity often preceding the onset of clinical spasms.^[2]^

First-line treatment consists of hormonal therapy, including adrenocorticotropic hormone (ACTH) or high-dose oral corticosteroids such as prednisolone.^[4–6]^ A paradigm shift in the management of WS followed the International Collaborative Infantile Spasms Study (ICISS), a large multicenter randomized controlled trial involving 377 infants across 5 countries. The study demonstrated that combination therapy with vigabatrin and hormonal therapy was significantly more effective than hormonal monotherapy.^[4]^ Further evidence refined treatment protocols, showing that very-high-dose prednisolone (8 mg/kg/day) achieved a 63% response rate as monotherapy, a marked improvement over conventional lower-dose steroid regimens.^[6]^ These findings emphasize the importance of early recognition and aggressive treatment because delays are consistently associated with poorer developmental and epileptic outcomes.^[4,7]^

EEG evaluation in WS has traditionally relied on subjective visual interpretation of hypsarrhythmia, an approach prone to inter-observer variability and lacking quantitative precision. Standardized low-resolution brain electromagnetic tomography (sLORETA) is an advanced quantitative EEG analysis method that enables 3-dimensional source localization using distributed modeling techniques.^[8]^ Based on the principle of maximal spatial smoothness, sLORETA assumes that neighboring neuronal populations exhibit similar activity patterns in both orientation and magnitude.^[9]^ This technique allows high-resolution spatial mapping of cortical activity and has been applied to various epilepsy syndromes for diagnostic assessment and treatment monitoring.^[10–12]^

Despite its potential, the application of advanced quantitative EEG source analysis, particularly sLORETA, in WS remains limited. Developing reliable quantitative biomarkers for WS could improve diagnostic accuracy, optimize therapeutic strategies, and provide deeper insights into its pathophysiology. Therefore, pre- and posttreatment EEG changes in WS patients receiving combination therapy with high-dose prednisolone and vigabatrin were investigated using sLORETA with the primary objective to quantitatively characterize treatment-related electrophysiological alterations and advance understanding of the underlying mechanisms of WS.

## 2. Methods

### 2.1. Study population

In the present study, 17 patients newly diagnosed with WS were prospectively enrolled between July 2020 and June 2025. Inclusion criteria were clusters of flexor, extensor, or mixed epileptic spasms; interictal EEG showing hypsarrhythmia; age at onset between 1 and 24 months; and clinical evidence of neurodevelopmental delay or regression.

### 2.2. Procedures

All patients underwent a baseline EEG prior to treatment initiation. Combination therapy was started with oral prednisolone at 8 mg/kg/day (administered in 3 divided doses; maximum, 60 mg/day) and vigabatrin at 100 mg/kg/day (administered in 2 divided doses). After 2 weeks, clinical and EEG reassessments were performed. Patients without adequate clinical or electrographic improvement, defined as persistent epileptic spasms or ongoing hypsarrhythmia, had their vigabatrin dose increased to 150 mg/kg/day, and prednisolone was gradually tapered over the subsequent 2 weeks. Follow-up EEG was performed at week 4 for all patients. This protocol was designed to systematically evaluate treatment-related electrophysiological changes.

### 2.3. EEG acquisition and data processing

EEG recordings were obtained using a NicoletOne™ EEG system with a sampling rate of 500 Hz over 30 minutes. Sleep was induced with chloral hydrate (50 mg/kg, with an additional dose up to 100 mg/kg if required). Twenty-one Ag/AgCl electrodes were positioned according to the international 10 to 20 system including Fz, Cz, Pz, A1, and A2, with additional channels for ocular and muscle artifacts, respiration, and electrocardiography. Electrode impedance was maintained below 5 kΩ, and filters were set between 1 and 70 Hz. Data were digitized at a 16-bit resolution.

For both baseline and week 4 EEGs, artifact-free 3-second epochs were manually selected from stage 1 and 2 sleep after visual inspection. Twenty epochs per patient per session were extracted, yielding a total of 680 epochs. Epoch selection was performed in a blinded manner by 1 investigator and independently verified by another. A fast Fourier transform was applied to each epoch; the resulting spectral data were exported as American Standard Code for Information Interchange files and imported into sLORETA software for further analysis.

### 2.4. sLORETA and statistical analysis

sLORETA was used to estimate current density distributions across 4 frequency bands: delta (1–4 Hz), theta (4–8 Hz), alpha (8–12 Hz), and beta (12–25 Hz). sLORETA computations employed a standardized 3-shell spherical head model (10,000 Monte Carlo simulations) with a regularization parameter (λ) of 0.1, 12-mm full-width at half-maximum smoothing kernel, and 6239 voxels at 5-mm^3^ resolution. The cortical model covered the cortical gray matter, hippocampus, and amygdala, mapped to Montreal Neurological Institute (MNI) coordinates and adjusted to Talairach space. Source localization utilized the standardized MNI brain template provided by sLORETA, without incorporating individual magnetic resonance imaging (MRI) data or additional electrode co-registration procedures.

Voxel-by-voxel comparisons of current density between pre- and posttreatment EEGs were performed using statistical nonparametric mapping (SnPM) with 5000 random permutations. Statistical significance was set at *P* <.01 and *P* <.05 corrected for multiple comparisons using the nonparametric method implemented in sLORETA.

## 3. Results

### 3.1. Clinical outcomes

Among the 17 enrolled patients, 9 were male and 8 were female, with a mean age at diagnosis of 6.8 ± 5.1 months (mean ± standard deviation). Nine patients were classified as symptomatic (7 with brain injury, 1 with tuberous sclerosis complex, and 1 with a congenital malformation), and the remaining 8 were categorized as cryptogenic or of unknown etiology.

After 2 weeks of combination therapy with prednisolone and vigabatrin, 5 patients (29.4%) achieved complete resolution of both epileptic spasms and hypsarrhythmia, and 4 patients (23.5%) demonstrated a >50% reduction in spasm frequency. The 12 patients without sufficient clinical or electrographic improvement underwent vigabatrin dose escalation to 150 mg/kg/day, with gradual prednisolone taper over the next 2 weeks.

At 4 weeks, hypsarrhythmia resolved in 12 patients (70.6%), confirmed based on follow-up EEG. Clinically, 11 patients (64.7%) achieved complete cessation of spasms, and an additional 3 (17.6%) showed a >50% reduction in spasm frequency.

### 3.2. sLORETA analysis

**Figure 1.**
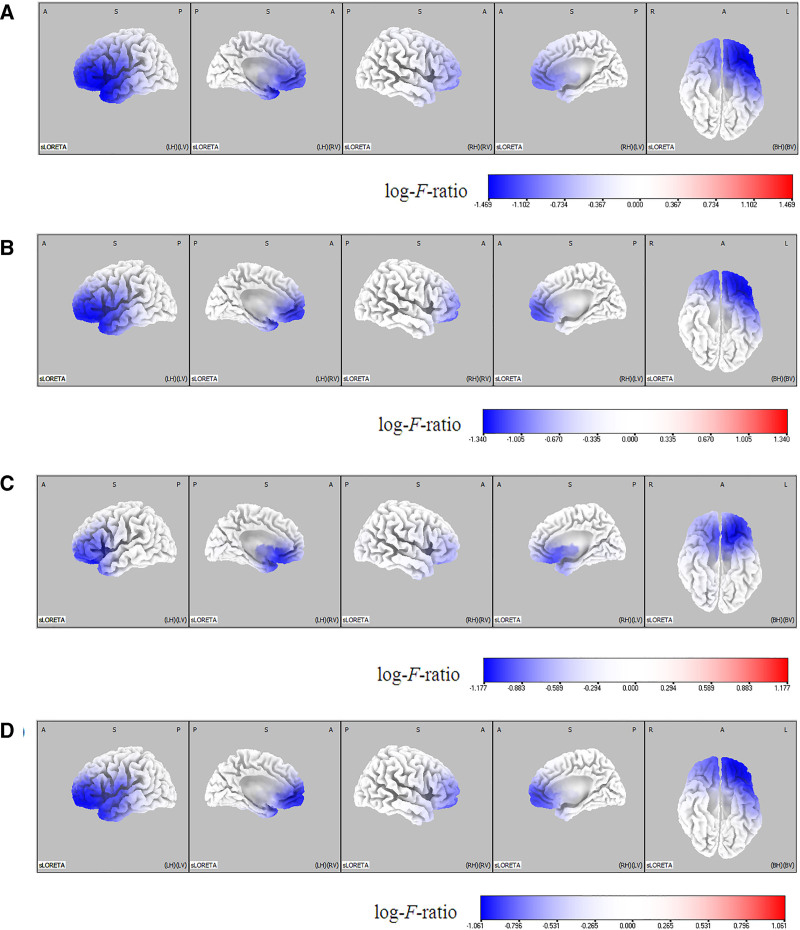
Statistical maps generated using sLORETA illustrate current density distributions across 4 EEG frequency bands projected onto a 3-dimensional fiducial brain cortex to compare pre- and posttreatment conditions. The panels show: (A) delta, (B) theta, (C) alpha, and (D) beta bands. SnPM analyses were conducted to evaluate differences in cortical current density between the 2 conditions. Log-*F*-ratio statistics were applied with color scales representing log-*F*-ratio values. Significance thresholds were set at log-*F*-ratio = ± 0.336, *P* <.05 and log-*F*-ratio = ± 0.398, *P* <.01. A = anterior, B = both, EEG = electroencephalography, H = hemisphere, L = left, P = posterior, R = right, S = superior, sLORETA = standardized low-resolution brain electromagnetic tomography, SnPM = Statistical nonparametric mapping, V = ventricle.

sLORETA analysis showed significant reductions in cortical current density across all frequency bands following treatment, with more notable decreases in the lower frequencies. Statistical thresholds were set at log-*F*-ratio = ± 0.336, *P* <.05 and log-*F*-ratio = ± 0.398, *P* <.01 (Fig. 1).

As summarized in Table 1, the 5 regions with the largest reductions in each frequency band were all localized to the left hemisphere, most frequently within the frontal lobe.

**Table 1:** Locations of the top 5 largest differences in current density of background EEG activity between pre- and posttreatment conditions.

	Hemisphere	Lobe	Log-*F*-ratio	*P*-value
Delta frequency band				
Inferior frontal gyrus	Left	Frontal	−1.47	<.01
Superior temporal gyrus	Left	Temporal	−1.47	<.01
Middle frontal gyrus	Left	Frontal	−1.46	<.01
Sub-gyral	Left	Frontal	−1.42	<.01
Insula	Left	Sub-lobar	−1.38	<.01
Theta frequency band				
Middle frontal gyrus	Left	Frontal	−1.34	<.01
Inferior frontal gyrus	Left	Frontal	−1.33	<.01
Superior temporal gyrus	Left	Temporal	−1.32	<.01
Superior frontal gyrus	Left	Frontal	−1.32	<.01
Sub-gyral	Left	Frontal	−1.30	<.01
Alpha frequency band				
Inferior frontal gyrus	Left	Frontal	−1.18	<.01
Middle frontal gyrus	Left	Frontal	−1.17	<.01
Extranuclear	Left	Sub-lobar	−1.16	<.01
Insula	Left	Sub-lobar	−1.16	<.01
Subcallosal gyrus	Left	Frontal	−1.15	<.01
Beta frequency band				
Middle frontal gyrus	Left	Frontal	−1.06	<.01
Inferior frontal gyrus	Left	Frontal	−1.05	<.01
Superior frontal gyrus	Left	Frontal	−1.05	<.01
Superior temporal gyrus	Left	Temporal	−1.03	<.01
Medial frontal gyrus	Left	Frontal	−1.03	<.01

EEG = electroencephalography.

**Figure 2.**
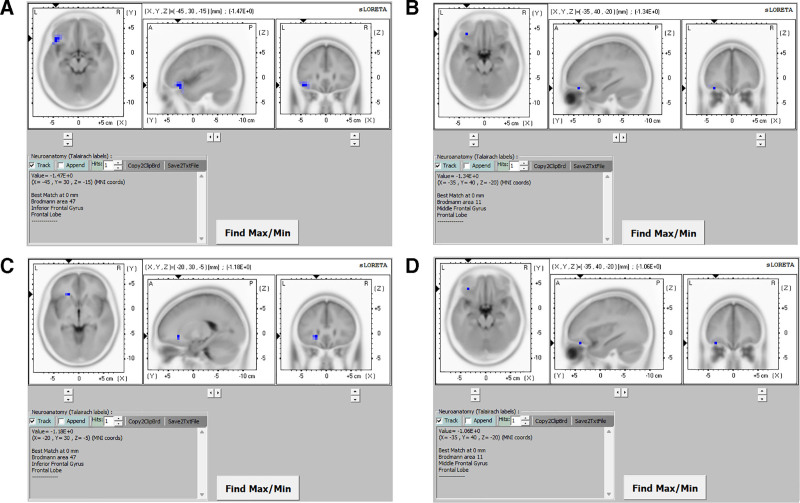
Statistical maps of the 4 EEG frequency bands (delta, theta, alpha, and beta) generated using sLORETA were projected onto a brain MRI template to compare pre- and posttreatment conditions. SnPM analyses were conducted to assess differences in current density distributions between the 2 conditions. The regions showing the greatest current density differences were (A) left inferior frontal gyrus for the delta band, (B) left middle frontal gyrus for the theta band, (C) left inferior frontal gyrus for the alpha band, and (D) left middle frontal gyrus for the beta band. A = anterior, EEG = electroencephalography, L = left, MNI coord = Montreal Neurological Institute coordinate, MRI = magnetic resonance imaging, P = posterior, R = right, sLORETA = standardized low-resolution brain electromagnetic tomography, SnPM = statistical nonparametric mapping.

**Delta band (1–4 Hz):** Greatest reductions were observed in the left inferior frontal gyrus (MNI coordinates [x, y, z = −45, 30, −15], Brodmann area 47; log-*F*-ratio = −1.47, *P* <.01; Fig. 2A), followed by the left superior temporal gyrus (log-*F*-ratio = –1.47, *P* <.01), middle frontal gyrus (log-*F*-ratio = −1.46, *P* <.01), sub-gyral region (log-*F*-ratio = −1.42, *P* <.01), and insula (log-*F*-ratio = −1.38, *P* <.01).

**Theta band (4–8 Hz):** The most significant reduction occurred in the middle frontal gyrus (MNI coordinates [x, y, z = −35, 40, −20], Brodmann area 11; log-*F*-ratio = −1.34, *P* <.01; Fig. 2B), followed by the inferior frontal gyrus (log-*F*-ratio = −1.33, *P* <.01), superior temporal gyrus (log-*F*-ratio = 1.32, *P* <.01), superior frontal gyrus (log-*F*-ratio = −1.32, *P* <.01), and sub-gyral area (log-*F*-ratio = −1.30, *P* <.01).

**Alpha band (8–12 Hz):** Largest decreases were observed in the inferior frontal gyrus (MNI coordinates [x, y, z = −20, 30, −5], Brodmann area 47; log-*F*-ratio = −1.18, *P* <.01; Fig. 2C), followed by the middle frontal gyrus (log-*F*-ratio = −1.17, *P* <.01), extranuclear area (log-*F*-ratio = −1.16, *P* <.01), insula (log-*F*-ratio = −1.16, *P* <.01), and subcallosal gyrus (log-*F*-ratio = −1.15, *P* <.01).

**Beta band (12–25 Hz):** This band showed the smallest changes, with the middle frontal gyrus ((MNI coordinates [x, y, z = −35, 40, −20], Brodmann area 11; log-*F*-ratio = −1.06, *P* <.01; Fig. 2D) most affected, followed by the inferior frontal (log-*F*-ratio = −1.05, *P* <.01), superior (log-*F*-ratio = −1.05, *P* <.01) and medial (log-*F*-ratio = −1.03, *P* <.01) frontal gyri, and superior temporal gyrus (log-*F*-ratio = −1.03, *P* <.01).

Overall, treatment-related reductions in cortical activity were most prominent in the left frontal cortex, with additional temporal and deep cortical involvement depending on frequency band.

## 4. Discussion

This study investigated electrophysiological changes in patients with WS treated with high-dose prednisolone and vigabatrin, using source-localized EEG analysis via sLORETA. Clinically, our remission rate (64.7%) aligns with outcomes reported in the ICISS trial, where 72% of infants achieved spasms cessation following combination therapy with high-dose hormonal therapy and vigabatrin.^[4]^ In the same trial, high-dose hormonal monotherapy showed a 57% response rate. Electro-clinical response–defined as both clinical cessation of spasms and resolution of hypsarrhythmia–was reported in 66% of the combination therapy group and 55% of the hormonal monotherapy group. In our study, 64.7% of patients achieved complete clinical remission, and 70.6% showed resolution of hypsarrhythmia at 4 weeks. These findings appear broadly consistent with the efficacy observed in the combination therapy arm of the ICISS trial, despite the absence of a comparator group. To further delineate the additive effects of corticosteroids, future randomized controlled trials comparing combination therapy with vigabatrin monotherapy would be valuable.

After 4 weeks of combination therapy, cortical current density showed consistent and significant reductions across all frequency bands, with the most pronounced decreases in the low-frequency range, particularly in the delta band. These reductions were most prominent in the frontal cortex, with additional involvement of temporal and deep cortical regions depending on the frequency band. These findings suggest that the therapeutic effects of combination therapy are associated with suppression of pathological cortical hyperexcitability in WS.

The marked decrease in low-frequency activity, especially within the delta band, corresponds to the hallmark EEG feature of hypsarrhythmia, namely high-amplitude, disorganized slow waves. This supports the hypothesis that treatment attenuates excessive neuronal activity underlying these pathological patterns and offers a measurable marker of improved cortical excitability.

Previous studies have also reported elevated neuronal activity across multiple frequency bands in WS, reinforcing the concept of widespread cortical dysfunction. For example, Burroughs et al found increased absolute power during sleep across all frequency bands in patients with infantile spasms compared with controls.^[13]^ Similarly, Smith et al reported elevated power in the delta, theta, and alpha bands during wakefulness^[14]^ and, in a separate study, observed significant increases across all bands in sleep EEGs.^[15]^ Although the specific frequency bands vary slightly among studies, broad consensus exists regarding diffuse cortical hyperexcitability. Our findings extend this consensus by demonstrating that combination therapy reduces cortical activity across all frequency bands, particularly in the low-frequency range.

Topographically, the most pronounced reductions in cortical current density were localized to the frontal cortex, with additional involvement of the temporal and deep cortical areas. This spatial pattern aligns with previous findings implicating the frontal lobes in the pathophysiology of WS. For example, Smith et al reported increased broadband amplitude in the frontal and central regions of patients with infantile spasms.^[14]^ Haginoya et al described a case of focal hypsarrhythmia originating from the right frontal cortex, accompanied by hyperperfusion on single photon emission computed tomography (SPECT) imaging.^[16]^ Similarly, Sztriha et al observed reduced perfusion in the bilateral anterior and mid-frontal cortices as well as in surrounding perisylvian areas compared with controls.^[17]^ Collectively, these findings emphasize the frontal cortex as a key region involved in the generation of hypsarrhythmic discharges.

However, posterior cortical regions have also been implicated. Jha et al noted that high-amplitude discharges often originate in the occipital or occipitotemporal regions before spreading anteriorly.^[18]^ Supporting this, coherence analysis by Japaridze et al identified the occipital cortex as a primary source of delta activity in hypsarrhythmia.^[19]^ Chiron et al further observed a mixed pattern of hyperperfusion in frontal areas and hypoperfusion in posterior cortices, suggesting functional contributions of both anterior and posterior regions.^[20]^ Taken together, these findings support the concept that hypsarrhythmia arises from complex and distributed network dysfunction. Our results further indicate that combination therapy may preferentially suppress hyperactivity in anterior cortical regions, potentially modulating abnormal cortico-subcortical network dynamics involved in the generation and propagation of hypsarrhythmia. While sLORETA focuses on cortical activity, a prior SPECT study in cryptogenic WS demonstrated hypoperfusion in the thalamus, hippocampus, and lenticular nucleus, as well as in multiple cortical regions.^[21]^ In contrast, a simultaneous EEG-functional MRI (fMRI) study reported increased blood oxygen level-dependent responses in the thalamus, hippocampus, and brainstem, during hypsarrhythmia bursts, suggesting complex cortico-subcortical dynamics.^[22]^ Future work integrating EEG-sLORETA with resting-state fMRI could elucidate cortico-thalamic dysconnectivity.

Notably, our study identified a frequency-dependent gradient in treatment response, with the most pronounced reductions in cortical current density occurring in the lower-frequency bands, particularly delta and theta. Furthermore, an anterior-dominant pattern was centered in the frontal lobe, indicating that the pathological activity associated with hypsarrhythmia is not uniformly distributed but rather spatially concentrated in specific cortical regions. Although hypsarrhythmia has traditionally been characterized as a chaotic and disorganized EEG pattern, these findings indicate that underlying regularities may exist, both in terms of frequency-specific and spatial distributions. This implies that the abnormal brain activity in WS may be differentially modulated across distinct neural networks, depending on both frequency and location. Understanding these frequency- and region-specific patterns of cortical modulation could provide novel insights into the neurophysiological mechanisms underlying hypsarrhythmia.

However, this study has several limitations. First, the relatively small sample size limits the generalizability of the findings and reduces the statistical power to perform correlation analyses between the effects of combination therapy and changes in cortical current density. Consequently, we could not determine whether reductions in EEG activity were directly associated with therapeutic outcomes. Such correlations would be critical for establishing quantitative EEG markers as reliable predictors of clinical response. Larger, multicenter studies are needed to validate these results and investigate such relationships in more detail. Second, while sLORETA provides valuable, unbiased cortical source localization focused on the cortical gray matter, hippocampus, and amygdala, it cannot assess deeper subcortical structures such as the thalamus and brainstem, which may play important roles in the pathophysiology of WS. Future studies should incorporate advanced neuroimaging modalities such as fMRI, SPECT, positron emission tomography, and magnetoencephalography (MEG) to better characterize the involvement of these deeper networks. Despite these limitations, the use of SnPM with voxel-wise randomization (5000 permutations) was appropriate for the sample size and yielded robust findings.^[9,23]^ However, replication with larger samples and complementary multimodal approaches are necessary to confirm and extend these results. Third, source localization in this study was performed using sLORETA’s built-in standardized MNI template, without the use of individual MRI data. While this approach facilitates practical group-level analysis, it may limit anatomical precision and spatial accuracy in source localization. Future studies employing individualized MRI data may further enhance localization fidelity and strengthen neuroanatomical interpretations.

In conclusion, this study demonstrates that high-dose prednisolone combined with vigabatrin significantly reduces cortical hyperexcitability across multiple frequency bands, particularly in the low-frequency range. Prominent effects were observed in the frontal and temporal lobes as well as deep cortical structures in patients with WS. These findings offer new insights into the pathophysiology of WS by highlighting a frequency-dependent gradient in treatment response and an anterior-dominant pattern. These findings suggest that therapeutic interventions can modulate pathological brain activity in a region- and frequency-specific manner. Future research should involve larger patient cohorts with long-term follow-up to explore correlations between clinical improvements and electrophysiological changes. Furthermore, the integration of multimodal neuroimaging techniques, such as fMRI, SPECT, and MEG, that can assess both cortical and subcortical structures, are needed to further elucidate underlying mechanisms and guide precision medicine approaches tailored to the pathophysiology of WS.

## Author contributions

**Conceptualization:** Jooyoung Lee, Tae-Hoon Eom.

**Data curation:** Jooyoung Lee, Ja Un Moon, Tae-Hoon Eom.

**Formal analysis:** Jooyoung Lee, Ja Un Moon, Tae-Hoon Eom.

**Methodology:** Tae-Hoon Eom.

**Resources:** Eu Gene Park, Il Han Yoo, Ji Yoon Han, Tae-Hoon Eom, Joong Hyun Bin.

**Supervision:** Tae-Hoon Eom.

**Writing – original draft:** Jooyoung Lee, Tae-Hoon Eom.

**Writing – review & editing:** Tae-Hoon Eom.
